# A Novel Liquid Biopsy Strategy to Detect Small Amounts of Cancer Cells Using Cancer-Specific Replication Adenoviruses

**DOI:** 10.3390/jcm9124044

**Published:** 2020-12-14

**Authors:** Masahiro Takakura, Emi Takata, Toshiyuki Sasagawa

**Affiliations:** Department of Obstetrics and Gynecology, Kanazawa Medical University, Ishikawa 920-0293, Japan; emi0415@kanazawa-med.ac.jp (E.T.); tsasa@kanazawa-med.ac.jp (T.S.)

**Keywords:** circulating tumor cells (CTCs), adenovirus, telomerase

## Abstract

Circulating tumor cells (CTCs) are a promising source of clinical and biological cancer information and can be a material for liquid biopsy. However, detecting and capturing these cells remains a challenge. Various biological factors (e.g., cell surface proteins, cell size, deformability, or dielectrophoresis) have been applied to detect CTCs. Cancer cells dramatically change their characteristics during tumorigenesis and metastasis. Hence, defining a cell as malignant using such a parameter is difficult. Moreover, immortality is an essential characteristic of cancer cells. Telomerase elongates telomeres and plays a critical role in cellular immortality and is specifically activated in cancer cells. Thus, the activation of telomerase can be a good fingerprint for cancer cells. Telomerase cannot be recognized by antibodies in living cells because it is a nuclear enzyme. Therefore, telomerase-specific replication adenovirus, which expresses the green fluorescent protein, has been applied to detect CTCs. This review explores the overview of this novel technology and its application in gynecological cancers.

## 1. Introduction

Pathological examination of tumor tissue is an important step in medical practice. However, there are inherent problems in this procedure. One is whether the target tissue is truly harvested, and the other is the invasiveness to the patient. Fine-needle biopsies or computed-tomography- or ultrasound-guided biopsies have been developed to overcome these problems, but they are not sufficient. Liquid biopsy is the collection of blood samples from patients, targeting circulating tumor cells (CTCs), circulating tumor DNA (ctDNA), circulating microRNA, exosomes, and so on. The analytical procedures for each material and their clinical value are currently being studied.

Analyzing the cancerous cells in the blood stream is especially important because blood-borne metastasis is the main reason for cancer death, and the intravasation of cancer cells and their spread through blood flow is a critical step in metastasis. Thus, CTCs in the peripheral blood have been considered a promising biomarker and a powerful source of biological information on cancer. However, distinguishing a small number of tumor cells from the numerous blood cells (as low as one CTC in 10^6^–10^7^ leukocytes) poses a challenge. Epithelial cell surface markers, such as epithelial cell adhesion molecule (EpCAM) or mucin 1 (MUC1), or cytokeratin, which are keratin-containing intermediate filaments expressed in the cytoskeleton of epithelial cells, have been applied for this purpose. This is represented by CellSearch™ system (Veridex, LLC, Warren, NJ, USA), which consists of immunomagnetic enrichment with EpCAM antibody and detection with positive cytokeratin (CK8/18/19) staining and 4′,6-diamidino-2-phenylindole (DAPI) as well as CD45 (leukocyte common antigen)-negative staining [1]. Other biological characteristics have also been applied such as cell size (CTCs are usually bigger than the surrounding hematic cells)-based microfluidic devices [2,3], cellular deformability [4], or electrokinetic-characteristic-employing dielectrophoresis [5].

Cancer has a heterogeneous cell population that changes appearance depending on the situation. Cells do not express epithelial markers, including EpCAM or cytokeratins, during epithelial-to-mesenchymal transition (EMT) associated with cancer progression and intravasation of cancer cells [6,7]. In terms of cell size, CTCs detected by fluorescence in situ hybridization-based technology based on chromosome 8 aneuploidy have also been reported to be smaller than the white blood cells (WBCs) and are associated with poor prognosis in hepatocellular carcinoma, suggesting that there is some degree of risk involved in excluding smaller cells [8]. In addition, it is sometimes difficult to distinguish living cells from dead ones based on epithelial markers or cell size alone. In this regard, epithelial markers or cell-size-dependent detection of CTCs is not a sufficiently valid procedure. Thus, the development of absolute cancer markers to reliably detect living CTCs is necessary to overcome the diversity and dynamic nature of cancer cells.

Immortalization is an unchanging feature of cancer cells. Telomeres, the terminal ends of linear chromosome, are shortened during cell division. The excess shortening of telomeres causes a halt in cell division (cellular senescence), owing to which normal cells do not proliferate forever. Telomerase is a nucleoprotein complex that functions as an RNA-dependent DNA polymerase. It elongates telomeres by adding TTAGGG repeats and is essential for cellular immortalization [9]. Human telomerase is activated in >85% of cancers regardless of its origin, whereas it is attenuated in almost all the somatic cells, suggesting that the activation of telomerase is a fingerprint of cancer cells [10,11]. Telomerase is a nuclear protein, and its expression is very low. Therefore, telomerase cannot be detected in living cells by methods such as fluorescent antibodies. Telomerase consists of three main components: RNA template (hTR) [12], telomerase-associated protein (hTEP1) [13], and human telomerase reverse transcriptase (hTERT) [14,15]. hTERT expression is the determinant of telomerase activity because hTR and hTEP1 are broadly expressed in most cells. hTERT expression is strictly regulated by its gene promoter [16,17,18]. Thus, the transcriptional activation of *hTERT* can be a hallmark of cancer cells. In this review article, we discuss the application of cancer-specific regulation of *hTERT* transcription in liquid biopsy to detect CTCs in gynecological cancers.

## 2. Detection of CTCs in Gynecological Cancer Using Conventional Technologies and Their Clinical Significance

### 2.1. Endometrial Cancer

The largest study on endometrial cancer was performed using the MetaCell^®^ (MetaCell s.r.o., Ostrava, Czech Republic), a size-based CTC-capturing device. Sixty-nine of 92 (75%) patients were positive for CTCs. There was no relationship between CTC positivity and clinicopathological findings such as stage of disease, tumor grade, or lymph node involvement [19].

CellSearch™-based CTC detection was performed in studies comprising a smaller number of patients. Bogani et al. detected CTCs in 2 of 28 (7.1%) patients with endometrial cancer, and it was associated with myometrial invasion and lymph node involvement [20]. Ni et al. also performed CTC detection in 40 patients, resulting in 6 positives (15%). The CTC positivity was associated with cervical involvement [21]. Lemech et al. detected CTCs in 18 of 30 (60%) patients, which was associated with tumor stage, size, non-endometrioid histology, and poor overall survival (OS) [22]. Casas-Arozamena et al. simultaneously analyzed CTCs and ctDNA. Fourteen of 36 (39%) patients were positive for CTCs. They were weakly associated with non-endometrioid histology, higher tumor grade, and recurrent disease, but did not reach statistical significance [23].

Obermayr et al. analyzed female patients with cancer (21 breast, 23 ovarian, 25 cervical, and 25 endometrial). The enrichment of the CTC fraction using OncoQuick^®^ (Greiner Bio-One GmbH, Frickenhausen, Germany), a centrifugation-based separation system, was followed by RT-PCR for six genes upregulated in cancers (*cyclin E2*, *Holliday junction recognition protein*, *epithelial membrane protein 2*, *Myelin And Lymphocyte Protein 2* (*MAL2*), *Peptidylprolyl Isomerase C (PPIC)*, and *solute carrier family 6 member 8*). The percentage of positivity for at least one gene in breast, ovarian, cervical, and endometrial cancers was 29%, 44%, 64%, and 19%, respectively [24]. They also applied the Parsortix^®^ System (ANGLE, King of Prussia, PA, USA), a microfluidic device based on cell size and deformability, followed by RT-PCR for *EpCAM*, *PPIC*, *tumor suppressor candidate gene 3*, and *MAL2*. The positive rates for primary and recurrent gynecologic cancers, including endometrial cancer, were 32% and 14%, respectively [25].

### 2.2. Cervical Cancer

To date, several studies have been conducted on cervical cancer. The largest study was conducted as part of the Gynecologic Oncology Group 240 study, a randomized phase III trial of the anti-angiogenic drug bevacizumab for patients with recurrent/persistent and metastatic cervical cancer. CTCs were analyzed by CellSearch™ on pre-cycle 1 and 36 days after. A total of 176 patients participated in the CTC analysis. CTCs were detected in almost all cases before therapy, and in 81% of cases after cycle 1. The decrease in CTCs over time was associated with a lower risk of death. Moreover, among groups with high CTCs, bevacizumab treatment was associated with a reduction in the risk of death and an increase progression-free survival (PFS), suggesting increased neovascularization and effectiveness of anti-angiogenic drug for such patients [26].

In another study, digital-direct-RT-PCR for human papillomavirus E6/E7 gene was applied to detect CTCs, resulting in three positives out of 10 patients with cervical cancer [27]. Wen et al. detected CTCs by the combination of negative selection for CD45 by using anti-CD45 antibody coated magnetic beads and chromosome 8 aneuploidy by immunofluorescence in situ hybridization. Thirty-four of 99 (34%) patients with cervical cancer were positive for CTCs. The CTC positivity was associated with poor disease-free survival [28]. Du et al. also applied the same method to 107 patients. Eighty-six patients were positive for CTCs, which was associated with shorter PFS [29]. 

### 2.3. Ovarian Cancer

More studies have been reported on ovarian cancer than on the other two diseases. The CellSearch™ system has been applied for several studies. Poveda et al. analyzed the relationship between CTC positivity and prognosis in patients with ovarian cancer who were treated with pegylated liposomal doxorubicin with/without trabectedin. Thirty-one of 216 patients (14%) were CTC positive, which was associated with poor prognosis [30]. A Gynecologic Oncology Group phase II trial for the efficacy and safety of the mTOR inhibitor temsirolimus and for the evaluation of CTCs in recurrent or persistent ovarian and primary peritoneal cancer was conducted. Nineteen of 43 (44%) patients were CTC positive in the pre-treatment status. CTC positivity was associated with a lack of treatment response, and high expression of M30, an apoptosis marker, was associated with longer PFS [31]. Liu et al. performed serial CTC analysis in 78 women with newly diagnosed or recurrent ovarian cancer before and during treatments. Thirty-two of 88 patients were CTC positive at initial status. Initial positivity of CTCs did not correlate with prognosis, and the change in serial CTCs did not correlate with response to therapy [32]. Banys-Paluchowski et al. studied 43 patients with ovarian, fallopian, and primary peritoneal cancer (23 primary, 20 recurrence). Eleven patients (26%) were positive for CTCs at baseline. Positive CTCs at baseline was associated with shorter PFS and OS. In addition, the presence of CTCs after three cycles of chemotherapy (positive in 7.8%) was also associated with shorter OS [33]. 

The AdnaTest (Qiagen, Hilden, Germany), which consists of enrichment by immunomagnetic beads and amplification of target genes by RT-PCR (*EpCAM*, *MUC1*, and *human epidermal growth factor receptor 2 (HER2)* for the AdaTest BreastCancer; *EpCAM*, *MUC1*, *cancer antigen 125 (CA125)*, and (*ERCC1*) for the AdnaTest OvarianCancer; *phosphoinositide 3-kinase alpha*, Akt serine/threonine kinase 2 *(Akt2)*, and *Twist* for the AdnaTest EMT-1), has been applied in several studies. Aktas et al. applied the AdaTest BreastCancer for ovarian cancer before surgery and/or after chemotherapy. The CTC-positive ratios before surgery and after chemotherapy were 19% and 27%, respectively [34]. Kuhlmann et al. applied the AdnaTest OvarianCancer to analyze CTCs in 143 patients with ovarian cancer. CTCs were observed in 14% patients and were associated with worse OS. Furthermore, *ERCC1*-positive CTCs were observed in 8% of patients, and this was associated with PFS as well as OS [35]. Chebouti et al. studied CTCs and *ERCC1*-positivity in 65 primary patients with ovarian cancer at baseline and after platinum-based chemotherapy. The CTC-positive ratio was 17% at baseline. *ERCC1*-positive CTCs were observed in 15% of patients and in 12% after chemotherapy. The presence of *ERCC1*-positive CTCs after chemotherapy was associated with platinum resistance and shorter PFS and OS [36]. They also analyzed both epithelial CTCs and EMT-like CTCs in 91 patients with ovarian cancer before surgery and in 31 matched patients after chemotherapy using the AdnaTest OvarianCancer and AdnaTest EMT-1. The positive ratios of epithelial CTCs before surgery and after chemotherapy were 18% and 14%, respectively, whereas those of EMT-like CTCs were 30% and 52%, respectively. In stage I–III patients, the presence of epithelial CTCs or *PIK3Kα*-positive EMT-like CTCs before surgery was associated with worse OS and PFS [37]. 

The MetaCell^®^ has also been applied to ovarian cancer in two studies by the same authors. Kolostova et al. studied 118 patients with ovarian cancer before surgery. CTCs were positive in 77 (65%) patients. The presence of CTCs was associated with the existence of ascites, peritoneal carcinomatosis, and residual disease. Furthermore, capturing by the MetaCell^®^ enabled short-term in vitro culture of the CTCs [38]. They then analyzed 56 patients with ovarian cancer and found that CTCs were positive in 58% of the patients. Captured CTCs were applied for gene expression analysis, resulting in significant upregulation of the following genes: *keratin-7*, *Wilms’ tumor suppressor gene1 (WT1)*, *EpCAM*, *CA125*, *MUC1*, *keratin-18*, and *keratin-19* [39].

Enrichment by density-gradient centrifugation followed by a multi-marker immunostaining (EpCAM, Epidermal growth factor receptor, HER2, MUC1, and cytokeratins) was performed by Obermayr et al. Overall, 102 patients were tested at baseline, and 78 patients were also examined after chemotherapy. The CTC-positive ratios at baseline and after chemotherapy was 27% and 7.7%, respectively. The presence of CTCs at baseline was associated with worse prognosis in patients whose tumors were completely resected [40].

Magnetic beads coated with antibodies for the epithelial markers EpCAM, HER2, and MUC1 were applied to enrich CTCs in samples from patients with ovarian cancer, which were followed by multiplex RT-PCR for six genes (*EpCAM*, *HER2*, *MUC1*, *WT1*, *P16*, and *paired box gene 8*). CTCs were detected in 98 of 109 (89%) patients at baseline. Alternatively, CTCs were positive in 92 of 102 (94%) patients after chemotherapy. The average numbers of CTCs at baseline and after chemotherapy were 264 and 314, respectively. The existence of EpCAM-positive CTCs was associated with shorter OS, whereas the expression of other genes in CTCs had no relationship with prognosis [41]. 

Microfluidic devices have been applied to isolate CTCs in recent years. A combination of devices coated with anti-EpCAM or anti-fibroblast activation protein alpha (FAPα) antibodies were applied for patients with several kinds of cancers including ovarian cancer. All cases (eight metastatic and three localized) were positive for CTCs. EpCAM-positive CTCs were most abundant in patients with metastatic ovarian cancer (65–680/mL) compared with other cancer species. The next generation sequencing analysis of genomic DNA of CTCs revealed that both EpCAM- and FAPα-positive CTCs contained the same missense somatic mutations in *TP53* and *cadherin-1* genes and other single nucleotide polymorphisms, suggesting that they had the same origin [42]. Lee et al. applied an electrically modulated CTC capture and release system, in which biotin-doped polypyrrole was electrochemically deposited onto a microfluidic device followed by assembling streptavidin and biotinylated anti-EpCAM antibodies. Fifty-three of 54 patients (98%) were positive for single-cell CTCs or CTC clusters. The number of CTCs was associated with shorter PFS and the presence of CTC clusters was associated with resistance to platinum-based chemotherapy. Isolated CTCs were successfully cultured ex vivo in two patients [43]. The CD-PRIME™ (Clinomics, Ulsan, Korea), a size-based centrifugal microfluidic device, was applied to 47 consecutive blood samples obtained from 13 patients with ovarian cancer. At baseline, 84% of patients were positive for CTCs; the number of CTCs was associated with treatment response and disease progression [44]. A size- and deformability-based isolation of CTCs using a tapered-slit filter was applied for 30 patients with advanced ovarian cancer. The CTC detection rate before and after operation was 77% and 57%, respectively. The presence of postoperative CTCs was associated with shorter PFS [45]. The technologies applied to the detection of CTCs in gynecological field are summarized in Table 1. 

## 3. The hTERT Promoter Activity as Hallmark of the Cancer Cells

**Figure 1:**
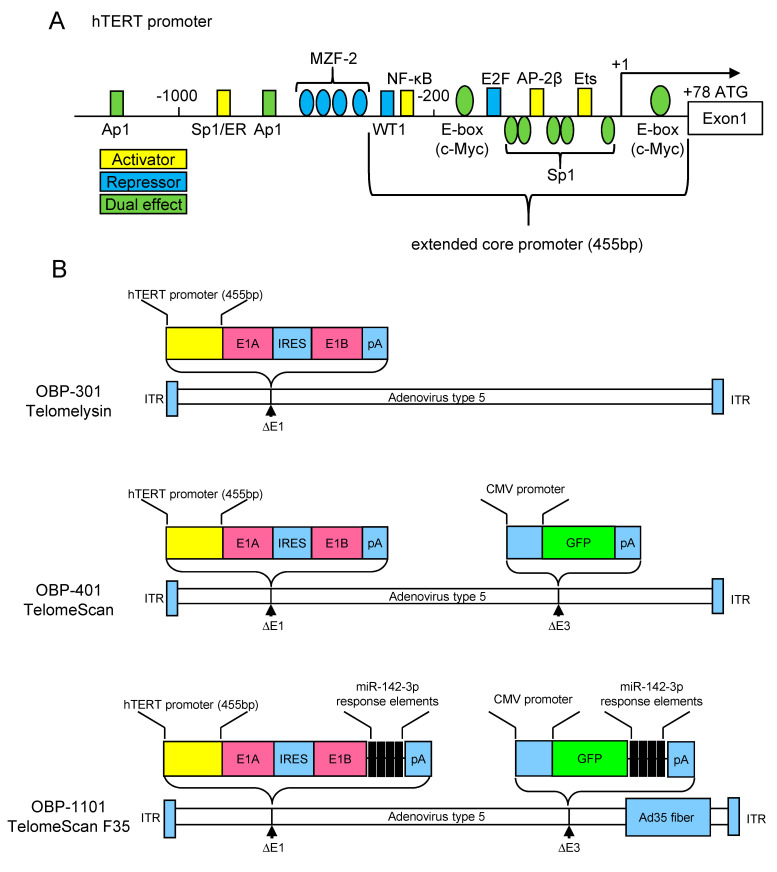
Structures of telomerase-specific replicating adenoviruses. (**A**) Representative transcription factor binding sites on the *human telomerase reverse transcriptase (hTERT)* promoter are shown. The sites on the promoter are not to exact scale. +1 indicates the transcription start site. ATG indicates the translation starting codon. (**B**) In OBP-301, the hTERT extended core promoter drives the expression of *E1A* and *E1B* genes linked with internal ribosome entry site (IRES). OBP-401 is a green fluorescent protein (GFP)-expressing variant, in which the cytomegalovirus promoter drives the expression of GFP inserted in the *E3* region. In OBP-1101, the adenoviral fibers were replaced with type 35 fibers to enable infection of coxsackievirus–adenovirus receptor-negative cells. The miR142-3p responsive elements were inserted into the 3′ untranslated regions (UTR) of the *E1* and *GFP* genes to attenuate non-specific GFP expression in blood cells. ITR, internal terminal repeat; pA, bovine growth hormone polyadenylation signal; AP1, Activator protein 1; SP1, Specificity protein 1; ER, estrogen receptor; MZF-2, myeloid Zinc-Finger Protein 2; NF-κB, nuclear factor kappa B; c-Myc, c-myelocytomatosis oncogene product; E2F, E2 promoter binding factor; AP-2, Activating enhancer-binding protein-2; Ets, erythroblast transformation specific proteins; GFP, green fluorescent protein; Ad35, adenovirus type 35.

*hTERT* is tightly regulated at the transcriptional level. Studies on the 5′-promoter of the *hTERT* gene has revealed that the proximal 260 base pairs function as the core promoter essential for cancer-specific transcriptional activation [16,17,18]. A schematic diagram of the *hTERT* promoter is presented in Figure 1A. Within the core promoter, there are two E-boxes, to which the heterodimers of the Myc family proteins bind. The c-Myc/Max heterodimer activates transcription; conversely, the Mad/Max heterodimer suppresses it [49,50]. Specificity protein 1 (Sp1) binds to five GC-box motifs in the core promoter and activates transcription in telomerase-positive cells [49,51]. On the contrary, Sp1 and Sp3 bind to their responsive sites to recruit histone deacetylase and suppress transcription in telomerase-negative normal cells [52]. These two-sided regulatory mechanisms of activation and repression via E-box and GC-box are most important for the transcriptional regulation of *hTERT*. In addition, an Ets-binding motif is located in the core promoter, which is responsible for transcriptional activity mediated by mitogen-activated protein kinase signaling pathways [53]. Activating enhancer-binding protein-2 (AP-2) was also identified as a transcriptional activator of the hTERT promoter [54]. E2 promoter binding factor (E2F) directly binds to the core promoter and suppresses transcription [55]. Nuclear factor kappa B (NF-κB) also activates hTERT transcription in both a direct and indirect manner [56,57]. WT1 suppresses hTERT transcription in both direct and indirect pathways [58]. 

The deletion mutant analysis of the *hTERT* promoter revealed that the proximal 455 base pair promoter (named extended core promoter; Figure 1A), which contains WT1 and NF-κB binding motifs in addition to the core promoter, has the strongest transcriptional activity in cancer cell lines [16]. The extended core promoter maintains its cancer specificity because the transcriptional repression mechanism was functional in normal cells [59]. 

## 4. Development of Telomerase-Specific Replication Adenovirus and Its Application for Diagnostic Use

Tumor-specific replicating adenovirus has been applied for cancer therapy in the last few decades. Oncolytic adenovirus was first described in 1996. Adenoviruses lacking the *E1B* gene were selectively replicated in P53-deficient tumor cells causing infected cell death [60]. The selective expression of the *E1A* gene was examined using tissue-specific promoters (e.g., *prostate-specific antigen* [61], *MUC1* [62], *osteocalcin* [63], *L-plastin* [64], *midkine* [65], and *E2F-1* [66]). However, these promoters are activated only in a limited type of tumors with transcriptional activities that are relatively low. The hTERT promoter has been applied to overcome these weaknesses. Adenoviruses that express the *E1A* gene under the control of the *hTERT* promoter exhibited efficient cancer-specific replication and cell lysis [67,68,69,70]. Kawashima et al. developed the modified adenovirus type 5 wherein both *E1A* and *E1B* genes were controlled by a single *hTERT* promoter resulting in improved cancer specificity (OBP-301, Telomelysin, Oncolys BioPharma Inc., Tokyo, Japan) [71]. The OBP-301 schematic diagram is shown in Figure 1B. Several clinical trials are underway to test the therapeutic effectiveness of OBP-301 for various cancers [72].

The cancer-cell-specific proliferative potential of OBP-301 has been considered useful for diagnostic purposes aside from its therapeutic aspect. The green fluorescent protein (GFP) gene was inserted into the OBP-301 to visualize infected cells (OBP-401, TelomeScan, Oncolys BioPharma Inc., Tokyo, Japan; Figure 1B). It was originally tested in a study of navigation surgery to visualize metastatic lymph nodes in mice models of colorectal cancer [73]. It was later applied to detect CTCs in the human blood samples. The advantages of this method are as follows. First, it is independent of cell size and cell surface markers. Second, it can avoid false positives in dead cells because OBP-401 does not infect dead cells. Third, it does not require dedicated machines. 

Thirty-seven cases of gastric cancer and several cases of other cancers (e.g., colon, hepatocellular, breast, and lung) were analyzed. The hemolyzed blood samples were infected with OBP-401 and observed using fluorescent microscopy after incubation [74]. This OBP-401-based detection method was also applied for breast [75], bladder [76], and non-small-cell lung cancers [77]. In addition, some modifications have been added to the original protocol. The CTCs in gynecological cancers were analyzed by adding CD45 staining to eliminate false-positive WBCs (described in more detail in Section 5) [46]. The enrichment of the CTC fraction using the OncoQuick^®^ was followed by OBP-401 infection in patients with high-grade glioma [78] and metastatic melanoma [79]. In another experiment, the cell size threshold was applied to the GFP-positive cells in gastric [80,81] and several kinds of cancers, including a small number of gynecological cancers [47].

However, the detection of CTCs using OBP-401 has several drawbacks. First, OBP-401 cannot effectively recognize coxsackievirus–adenovirus receptor (CAR)-negative CTCs because OBP-401 was constructed based on the adenovirus type 5 that recognizes CARs as infection receptors. In addition, it is reported that CAR expression is suppressed during the epithelial–mesenchymal transition (EMT) [82]. Therefore, the effective infection of CAR-negative or low-expressing cells with viral vectors was considered necessary. Second, a small fraction of blood cells has telomerase activity which causes a false-positive GFP signal [83,84]. OBP-1101 (TelomeScan F35, Oncolys BioPharma Inc., Tokyo, Japan), a second-generation telomerase-specific replicating adenovirus, was generated by modifying OBP-401 to overcome these weaknesses [85]. Adenovirus type 5 fibers were exchanged for type 35 fibers, which bind to CD46 in OBP-1101. Thus, OBP-1101 can effectively infect CAR-negative cells because CD46 is universally expressed in most human cells [86]. The miR-142-3p complementary sequences were inserted into the 3′-untranslated regions (UTR) of the *E1* and *GFP* genes to prevent false-positive signals in the blood cells. Inserting the miR-142-3p complementary sequence in the 3′-UTR of the target genes enables the suppression of blood cell expression in a post-transcriptional manner because it is a blood-cell-specific microRNA (Figure 1B). OBP-1101 was applied in non-small-cell lung cancer, resulting in the remarkable regression of false-positive cells [85] and the successful detection of both EMT and non-EMT CTCs [87]. This was applied in cervical cancer, which also resulted in the detection of EMT CTCs (described in more detail in the next chapter) [48].

The Ad5GTSe, a telomerase-specific replicating adenovirus driven by the hTERT promoter, was developed by another group. Furthermore, the hTERT promoter was modified by inserting repressor elements into it. The Ad5GTSe was applied to detect CTCs in breast and pancreatic cancers, resulting in a decrease in false-positive signals in the blood cells [88]. In the other report, the herpes simplex virus–based oncolytic virus vector wherein the endogenous infected cell protein 4 promoter was replaced with the hTERT promoter was also modified to express GFP. This was used to detect the CTCs in various types of cancers (lung, colon, liver, gastric, pancreas, and glioma; 326 cases in total) [89].

## 5. Detection of Telomerase-Specific Replication Adenovirus to Detect CTCs in Gynecological Cancers

**Figure 2:**
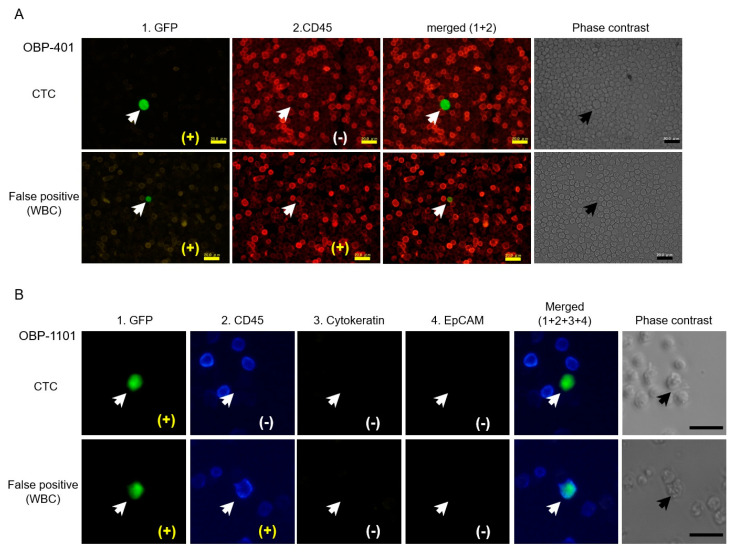
Representative pictures of CTCs detected by telomerase-specific replicating adenoviruses; (**A**) OBP-401 was infected with the blood samples from a patient with cervical cancer (top row) and a healthy volunteer (bottom row). GFP(+)/CD45(−) cells were recognized as CTCs, and GFP(+)/CD45(+) cells were false-positive blood cells. (**B**) OBP-1101 was infected with the blood samples from patients with cervical cancer. As with OBP-401, CTCs were GFP(+)/CD45(−) and false-positive cells were GFP(+)/CD45(+). The observed CTCs were negative for the epithelial markers EpCAM and cytokeratin.

Three reports have been published to date about the detection of CTCs in gynecological cancers using the telomerase-specific replication adenovirus (Table 2). As briefly aforementioned, the OBP-401 was applied to detect CTCs in patients with gynecological cancer. The viral load was increased to improve sensitivity compared with the original protocol. Consequently, false-positive WBCs also increased in preparatory experiments. Therefore, CD45 immunostaining was added, and the GFP-positive/CD45-negative cells were recognized as having tumor origin (Figure 2A). CTCs were detected in 21 of 53 patients (40%). This included 10 of 19 cervical cancers (56%), 5 of 17 endometrial cancers (30%), 5 of 14 ovarian cancers (36%), and 1 of 4 vulvar/vaginal cancers (25%). The average number of CTCs in 5 mL of blood was 2.9 (range, 1–10). No significant difference was evident in the CTC-positive ratio, the number of CTCs among cancer types, clinical stages, or other clinicopathological findings. However, the serial experiments during cancer treatment have shown that residual CTCs after treatment were associated with poor response to treatment, indicating that CTCs can be a surrogate marker of treatment response [46].

Yabusaki et al. analyzed the normal blood-infected OBP-401. The immunocytological staining of GFP-positive blood cells revealed that they were the CD13- and CD14-positive monocytes. The average and standard deviation of these cells were 7.18 and 0.65 µm, respectively, with a set size threshold of 8.4 µm to exclude the GFP-positive monocytes with a 95% confidence interval. This criterion was applied to several types of cancer, including four endometrial (three positive CTCs) and eight cervical (two positive CTCs) cancers. However, the sample size was too small to find an association with clinicopathological findings [47].

The CTCs in patients with uterine cervical cancer were detected using the second-generation telomerase-specific replicating adenovirus, OBP-1101. CTCs were detected in six of 23 cervical cancer cases (26%). The average number of CTCs in each sample was 2.5 (range, 1–4). No significant difference was noted between the positive ratio of the CTCs and number of CTCs compared with previous experiment using OBP-401 conducted by the same authors. The average and standard deviation of the number of false-negative cells (GFP-positive/CD45-positive) were 4.57 and 9.25 (range, 0–43), respectively, which were significantly fewer than in experiments using OBP-401. The presence of CTCs did not correlate with any clinicopathological factors. Immunostaining for epithelial markers (EpCAM and/or cytokeratin) was also performed for CTCs (Figure 2B). However, all CTCs were negative for both EpCAM and cytokeratin, suggesting that these cells were in EMT status. In five of six CTC-positive cases, human papillomavirus (HPV) DNA of the same subtype as the corresponding primary lesion was identified, indicating that CTCs were indeed derived from HPV-infected cancer [48].

These studies all had a small number of participants and thus were not sufficient to conclude clinical usefulness. However, the simplicity and certainty of a telomerase-specific replicating adenovirus to detect living CTCs are notable.

## 6. Conclusions

The development of novel techniques for the detection of CTCs using conditionally replicating adenoviruses is still in its infancy. Telomerase activation is a hallmark of cancer and is associated with EMT and tumor metastasis. In terms of applying this feature as an indicator of cancer cells, telomerase-specific replicating adenovirus, and especially its modified version OBP-1101, is promising. It enables the detection of CTCs independent of epithelial cell surface markers and cell size while excluding dead cells. Additionally, it has been suggested that hTERT has multiple biological functions independent of telomere lengthening. hTERT overexpression is associated with EMT and cancer stemness [90,91]. Thus, it is possible that the telomerase-specific replicating adenovirus preferentially detects CTCs in the EMT status, which was suggested in the study on cervical cancer using OBP-1101 [48]. It has been suggested that CTCs detected by OBP-401 can be therapeutic biomarkers. Therefore, serial examinations during cancer treatment would be of benefit.

Several factors need to be addressed. All the studies that applied OBP-401 or OBP-1101 had a limited number of samples. The cost and effort required for sample processing is not so much, but the application of fluorescence-activated cell sorting or digital PCR may be necessary to make it more efficient for larger-scale studies or clinical applications. The positive ratio of CTCs using OBP-401 or OBP-1101 was approximately 30–40%, which is somewhat low to be used for screening or initial diagnosis of tumors. There are several possible reasons. Telomerase is negative in approximately 10% of cancers. Furthermore, it has also been reported that there are alternative pathways to enhance *hTERT* transcription apart from activating the wild-type *hTERT* promoter. The point mutations in the *hTERT* promoter can induce *hTERT* overexpression by creating de novo Ets-binding motifs [92,93]. The increase in *hTERT* copy number has also been reported to upregulate *hTERT* gene expression [94,95]. When relying solely on the activation of the *hTERT* promoter, the CTCs may be missed in these tumors. To prevent this, a combination of another cancer-specific promoter and *hTERT* promoter may be the solution. The numbers of CTCs detected by OBP-401 or OBP-1101 were fewer than those detected by CellSearch™ or other methods that utilize cell surface markers. However, the purity of true tumor cells isolated by this method was proven to be very high. The authenticity of detected CTCs should be proven by applying recently developed single-cell analysis technology to establish a gold standard for CTC detection. Furthermore, large-scale clinical studies are also highly anticipated in the near future.

The advantage of CTCs when compared with ctDNA or cfRNA as a liquid biopsy is that they contain information about the multi-omics of a tumor such as epigeomics or proteomics. The technology to analyze multi-omics in single-cell is under development [96]. The integration of this information is of great benefit in understanding the biology of cancer and establishing personalized medicine.

## Figures and Tables

**Table 1:** Circulating tumor cell (CTC) isolation methods applied for gynecological cancer.

Enrichment Method		Detection Method	Description	References
CellSearch™	Immunomagnetic beads (anti-EpCAM)	Cytokeratin (+)/DAPI (+)/ CD45 (−)	FDA approved Automated system	[20,21,22,23,26,30,31,32,33]
MetaCell^®^	Size based	RT-PCR	Commercially available kits Recoverable living cells	[19,38,39]
AdnaTest	Immunomagnetic beads	RT-PCR	Commercially available kits Specialized for target organ	[34,35,36,37]
Density-gradient centrifugation	Including OncoQuick^®^	RT-PCR, immunofluorescence	Recoverable living cells	[24,40]
Digital RT-PCR	Direct detection	RT-PCR (*E6*/*E7*)	No necessity of enrichment	[27]
WBC depletion	Immunomagnetic beads (anti-CD45)	Immunofluorescence (chromosome 8 aneuploidy)		[28,29]
Immunomagnetic beads	Anti-EpCAM, -HER2, -MUC1	RT-PCR		[41]
Microfluidic device	Anti-EpCAM, -FAPα	Next-generation sequencing	Recoverable living cells	[42,43]
Microfluidic device	Size and/or deformability based	RT-PCR, immunofluorescence	Recoverable living cells	[25,44,45]
Telomerase-specific replication adenovirus	Expressing GFP	Immunofluorescence, PCR (*E6*/*E7*)	Infect only living cells Independent of surface markers	[46,47,48]

EpCAM, epithelial cell adhesion molecule; WBC, white blood cell; MUC1, mucin 1; FAPα, fibroblast activation protein alpha; GFP, green fluorescent protein.

**Table 2:** Clinical studies about CTC detection using telomerase-specific replication adenoviruses.

Author	Year	Methodology	Subjects	CTCs (+) Rate	CTCs Number	Remarks
Takakura et al. [46]	2012	OBP-401 + CD45	19 cervical 17 endometrial 5 ovarian 4 vulvar/vaginal	40%	2.9/5 mL	Change in CTCs was a surrogate marker of treatment response.
Yabusaki et al. [47]	2014	OBP-401 + size threshold	8 cervical 4 endometrial	42%	Not available	Cells >8.4 µm and GFP (+) were recognized as CTCs.
Takakura et al. [48]	2018	OBP-1101 + CD45	19 cervical	26%	2.5/7.5 mL	CTCs were negative for epithelial markers and were proved of tumor origin by HPV PCR.

HPV, human papillomavirus.
